# 3.D. Workshop: An urgent need to fully understand Long Covid-19 and its sequela

**DOI:** 10.1093/eurpub/ckac129.135

**Published:** 2022-10-25

**Authors:** Organised by: EUPHA-CHR, Chair persons: Sarah Cuschieri (Malta), Julian Mamo (EUPHA-CHR)

## Abstract

**Introduction:**

Covid-19 has been a pandemic for the past two years. From early on, it became obvious that some of the individuals surviving the infection continued to experience symptoms beyond the acute phase of the infection or else developed symptoms after the acute infection. Multiple terminologies describing this phenomenon started to appear with ‘Long Covid’ being a popular nomenclature. It soon became evident that Long Covid can affect almost all the body's systems with a plethora of associated symptoms, while new symptoms keep on emerging across time. These persistent symptoms are noted to act differently among different individuals, irrelevant whether they were previously healthy or previously suffering from any chronic disease/s. Indeed, while some experienced persistent symptoms for a couple of weeks, some had persistent symptoms for months. The variations in the severity of symptoms are another feature that became evident among Long Covid sufferers. The year 2021 saw the approval of different Covid-19 vaccines and the initiation of vaccination rollouts across Europe. As the vaccine population coverage progressed, it was anticipated that the lower viral infectivity rate will also decrease the occurrence of Long Covid-19 among the vaccinated as compared to the unvaccinated. Yet breakthrough Covid-19 infections still occurred along with the development of Long Covid-19 among vaccinated, especially as new variants emerged and immunity waned. Long Covid-19 is still a relatively new condition with unspecified pathophysiology and unknown long-term disability trajectory. Therefore, it is imperative that this condition is put into the spotlight to comprehend this pandora's box while trying to prevent its occurrence and the associated sequela.

**Aim:**

Considering the fluidly of this condition with speculations that Long Covid might be the new chronic disease of this decade, this workshop is set to provide a multidisciplinary platform for emerging evidence on Long Covid originating from across Europe. In fact, the presentations in this workshop will tackle various aspects pertaining to Long Covid. The first presentation will provide evidence on the different risk factors and symptoms of Long Covid. The second presentation will discuss the association of multimorbidity and socio-economic factors as risk factors of Long-Covid. The third presentation will bring forward the perspectives of those suffering from the condition and the need for an integration of health care targeting both Long Covid and chronic diseases. While the fourth presentation will discuss the burden of Long-Covid symptoms and its impact on the quality of life. Finally, the bidirectional relationship between Long Covid-19 and NCDs along with the required public health action will be discussed. This will be followed by a discussion between the presenters and the audience.

**Key messages:**

• Long Covid-19 is a common occurrence among healthy and chronic diseases population alike with a plethora of contributing risk factors.

• An integrated healthcare plan is required to decrease the impact of Long Covid-19 on the population while simultaneously managing other underlying conditions and diseases.

